# Influence of the Cooling Temperature on the Surface Quality in Integrated Additive and Subtractive Manufacturing of Aluminum Alloy

**DOI:** 10.3390/ma17225496

**Published:** 2024-11-11

**Authors:** Jie Huang, Xiaolin Zhang, Zijue Tang, Qianglong Wei, Kaiming Hu, Ming Lou, Li Yan, Yawei Hu, Guoshuang Cai, Huan Qi, Yi Wu, Haowei Wang, Hongze Wang

**Affiliations:** 1 Shanghai Aircraft Manufacturing Co., Ltd., Shanghai 201324, China; 2 State Key Laboratory of Metal Matrix Composites, Shanghai 200240, China; 3 School of Materials Science & Engineering, Shanghai Jiao Tong University, Shanghai 200240, China; 4 School of Mechanical Engineering, Shanghai Jiao Tong University, Shanghai 200240, China; 5 School of Electrical Engineering and Automation, Anhui University, Hefei 230601, China; yaweihu@126.com; 6 Nanjing Huirui Photoelectric Technology Co., Ltd., Nanjing 211121, China; 7 Institute of Alumics Materials, Shanghai Jiao Tong University, Huaibei 235000, China

**Keywords:** additive manufacturing, directed energy deposition, blue laser, subtractive manufacturing, temperature

## Abstract

The surface quality of parts processed by laser additive manufacturing, especially laser-based directed energy deposition (LDED), makes it difficult to meet actual use requirements. In addition, defects generated during the long-term additive manufacturing process need to be removed in time. Therefore, laser additive and subtractive manufacturing is of great significance for additive manufacturing. The main difference between laser additive-subtractive manufacturing and pure subtraction is that a cooling temperature is required due to the laser process. Therefore, this work studies the temperature variation regularity during LDED and the milling processes, as well as the surface roughness, cross-sectional microstructure, and tool wear under different cooling temperatures for milling. The results show that there is a “turning point temperature” in LDED, and the value of the turning point temperature gradually increases with heat accumulation, which affects the initial temperature of the subtractive manufacturing. When subtracting, a high initial temperature improves surface quality and reduces tool wear, but an excessively high temperature will cause the aluminum alloy to adhere to the tool. Then, the smear metal is difficult to effectively remove, deteriorates the milling quality, and aggravates tool wear. It is found that the higher the cooling temperature generated, the wider the thermally insulated shear band. The insulated shear band may affect the quality of the additive and subtractive manufacturing. Finally, it is determined that the milling temperature of aluminum alloy in this work condition is about 100 °C.

## 1. Introduction

Additive manufacturing is a rapid prototyping technology with high design complexity and degrees of freedom. Directed energy deposition (DED) technology is widely used in industrial manufacturing due to its fast forming speed [1]. During the layer-by-layer repeated printing process of DED, unreasonable printing parameters and complex temperature field evolution in multilayer deposition can easily lead to poor deposition accuracy [2,3]. Poor interlayer bonding and material adhesion can also lead to the formation of defects such as cracks and pores [4]. Therefore, subtractive post-treatment has become an indispensable step before using DED-deposited parts [5]. Weiss et al. [6] first proposed an integrated additive and subtractive manufacturing (IASM) technology to eliminate excess materials in the additive manufacturing process and improve the forming quality. With the development of this technology, its manufacturing process includes two types: alternating between additive manufacturing and subtractive manufacturing, and subtractive manufacturing after printing [7]. At present, this technology has been used to achieve high-precision and high-efficiency production of printed products [8], and it is a potential manufacturing strategy to reduce material waste, meet customization requirements, and shorten construction periods [9,10].

In the process of additive manufacturing, the surface quality of the workpiece, the accuracy difference of the IASM machine, and the interlayer connection situation are the main issues worth studying. Du et al. [11] combined laser powder bed fusion (LPBF) technology with precision milling technology. Compared to LPBF technology alone, the surface quality and dimensional accuracy of mixed manufactured parts were greatly improved. DED is more flexible in the integration of subtractive processing [5]. The combination of DED and subtractive processing is beneficial for eliminating defects and improving surface quality [3]. Xie et al. [12] studied the coupling effect of additive and subtractive parameters on the forming quality. By combining experimental and numerical simulation techniques, the optimal range of IASM parameters for stainless steel aviation bearing brackets was obtained. The surface accuracy is jointly affected by parameters such as feed rate and input energy of additive manufacturing, subtractive feed rate, and axial cutting depth [13,14]. Currently, extensive research has been conducted on the parameters involved in material reduction processes. Bai et al. [15] have demonstrated that fluctuations in cutting force, which occur during chip separation at low cutting speeds, adversely affect surface quality. Conversely, an increase in cutting speed correlates with improvements in both surface quality and processing efficiency. However, the rapid dissipation of heat generated through high-speed contact between the cutting tool and the material limits the softening effect on the material, resulting in significant tool wear.

To achieve precise control over the surface quality of machined components, extend the service life of subtractive cutting tools, and reduce processing duration, techniques involving localized heating prior to material removal have been implemented. It has been reported that as the temperature rises from ambient to 437 K, the cutting forces experienced in the machining of AA6061 are reduced by up to 39% [16]. The softening effect induced by localized heating in the cutting zone is regarded as a pivotal factor contributing to a reduction in cutting forces and the enhancement of tool longevity [17]. The selection of an appropriate preheating temperature is intricately linked to the material properties. However, it is crucial to note that while localized heating diminishes cutting forces, the accompanying energy consumption and processing costs may hinder its widespread application. In additive manufacturing processes, the formation of a molten pool results in elevated workpiece temperatures [18]. Hence, implementing material reduction prior to the cooling is considered a more economical approach. Given that the entire workpiece undergoes heating, the resulting thermal field differs from that achieved through localized heating, thereby exerting a significant influence on the milling process. Li et al. [19] investigated the effect of sample temperature on the subtractive milling force of deposited titanium alloys. It is found that when the sample temperature exceeds 300 °C, the subtractive milling force gradually decreases with an increase in sample temperature, which is beneficial for reducing surface stress, lowering cutting energy consumption, and improving the service life of cutting tools [16,20]. However, for the IASM of Al alloys, the strategy for determining relevant parameters may be more complex [21]. Under a low sample temperature during the subtractive manufacturing process, a large cutting force may affect the surface quality [22]. Under a high intervention temperature, the low melting point characteristic of Al can easily cause material softening, affecting the surface microstructure and properties. Excessive adhesion of processing materials to cutting tools can also make it difficult to continue the subtractive manufacturing process [23]. However, the mechanism of influence of Al alloy temperature change on the surface quality of cutting samples and tool wear has not been revealed. Nonetheless, the mechanisms by which fluctuations in Al alloy temperature affect the surface quality of cutting samples and tool wear remain to be elucidated.

This work investigates the influence of the cooling temperature on the IASM of Al alloy. First, the temperature cooling characteristics of Al alloy laser additive manufacturing were analyzed. Second, the influence of different cooling temperatures on surface quality and cutting tools was investigated. Finally, the initial cooling temperature selection for the subtractive manufacturing process was discussed.

## 2. Materials and Procedure

### 2.1. Materials

Commercial AlSi7Mg (FCA101X-2, Xiangbang Composite Material, Anhui, China 55~100 um) was used as aluminum alloy powder in additive manufacturing. Table 1 summarizes the chemical compositions of the powder. The substrate was AlSi7Mg with a rectangular shape (145 mm × 145 mm × 20 mm).

### 2.2. Experimental Setup

In this work, the additive manufacturing and subtractive manufacturing equipment were separated. A heating platform was applied to connect additive and subtractive processes. The additive manufacturing equipment [21] was a blue laser-directed energy deposition (BL-DED) system which consisted of a blue laser generator (LDMblue 2000-60 VG7H, Laserline GmbH, Mülheim-Kärlich, Germany), a three-axis displacement platform, a laser deposition head (OTS-5 blue optics, Laserline GmbH, Mülheim-Kärlich, Germany), and a metal powder feeder (RC-PGF-D, Raycham Ltd., Nanjing, China). The energy was transferred from the blue laser generator to the laser deposition head by an optical fiber. The metal powder was delivered into a molten pool through a coaxial ring powder nozzle. The distance between the laser head and the substrate was 11 mm, while the defocusing amount of the powder flow was 1 mm. The laser beam was focused at 11 mm with a diameter of about 1.5 mm. Argon with 99.999% purity was applied as the shielding and carrier gas. The subtractive manufacturing equipment was a commercial milling device (NV-850, AWEA, Taiwan, China). The milling cutter was a carbide end mill with 4 edges and a 12 mm cutter edge diameter.

### 2.3. Experimental Procedure

**Figure 1:**
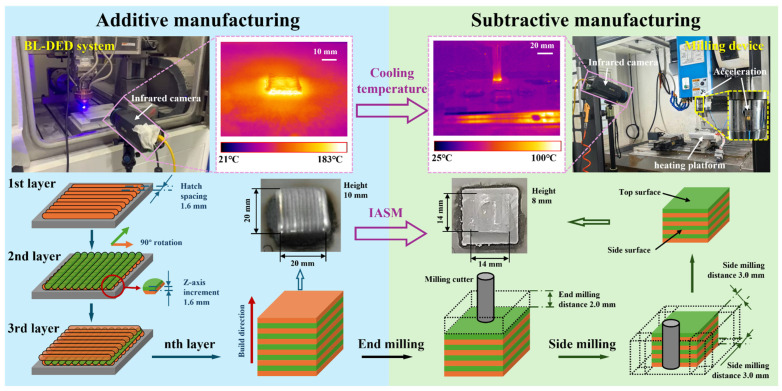
Photographs of the additive manufacturing and subtractive manufacturing equipment and samples with the monitoring devices.

The additive manufacturing process used the optimized process parameters found in previous works [21,24], which are shown in Table 2. The laser power, scanning speed, and powder feed rate were 1600 W, 18 mm/s, and 3 g/min, respectively. The hatch spacing of two tracks was 1.6 mm and the residence time between tracks was 0 s. The Z-axis increment was set at 0.3 mm and the residence time between layers was 30 s. The additive manufactured sample dimension was 20 mm × 20 mm × 10 mm. The shielding and carrier gas rates were set at a constant value of 600 L/h and 240 L/h, respectively. The subtractive manufacturing also used optimized process parameters for aluminum alloy, as seen in Table 3. The initial cooling temperature was set to 25 °C (room temperature), 100 °C, 200 °C, and 300 °C. The feed speed, spindle speed, and milling depth were 1000 mm/min, 3000 r/min, and 0.3 mm/layer, respectively. The samples of additive and subtract manufacturing are shown in Figure 1.

### 2.4. Monitoring and Characterization Methods

An infrared camera (A615, FLIR Systems Inc, Wilsonville, OR, USA) with a wavelength range of 7.5~14 μm and a temperature measurement range of −40~2000 °C was applied to monitor the temperature during both additive and subtractive manufacturing. The capturing parameters were 200 fps and 0.3 Mpix. The emissivity value was set to 0.65 based on the thermocouple temperature measurement.

An acceleration (TRIAXIAL 356A02, PCB, New York, NY, USA) was applied to measure the vibration signals with a sampling frequency of 5 KHz. The acquisition card was from Müller BBM in Germany.

The subtractive surface was inspected by a laser scanning confocal microscope (VK-X3000, KEYENCE Co., Ltd., Shanghai, China) to obtain color images and height images. The samples after subtractive manufacturing were cut by electron discharge machining to examine the cross-section including the milling surface. The cross-section of the sample was ground by 80 to 2500 grade and was polished using 5.0, 1.5, and 0.5 μm diamond suspension solutions. Then, the sample was etched with Kohler’s reagent. A metallographic microscope (Axio Imager.A2m, Carl Zeiss Microscopy GmbH, Jena, Germany) was used to observe the cross-section. The metallographic structure was analyzed by a Scanning Electron Microscope (SEM) (MAIA3, TSCAN China, Ltd., Shanghai, China) equipped with an energy-dispersive X-ray spectrometer (EDS).

## 3. Results

### 3.1. Variation Tendency of Temperature During Additive Manufacturing

**Figure 2:**
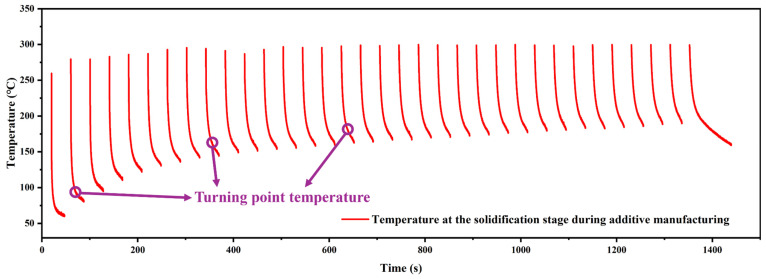
Temperature variation trend at the solidification stage during the additive manufacturing process.

Figure 2 shows the temperature trend between every layer. There are 33 layers. The results show that when there is no additional cooling, the heat continues to accumulate. After the first laser, the temperature of the substance rapidly rises to above 50 °C. After 5 layers, the temperature reaches about 130 °C. Then, during the following 28 layers, the temperature gradually increases to near 200 °C. In the end, under the condition of natural cooling, the temperature of the layer is still maintained above 150 °C after two minutes and above 100 °C after five minutes. Under the condition of natural cooling, the temperature falls more and more slowly. Moreover, for the DED, which claims to heat up and cool down quickly, the rapid temperature drop is only effective in some temperature intervals, and the intervals move upward with an increase in heat accumulation, which makes the bottleneck temperature of the rapid temperature drop higher and higher.

### 3.2. Variation Tendency of Temperature During Subtractive Manufacturing

**Figure 3:**
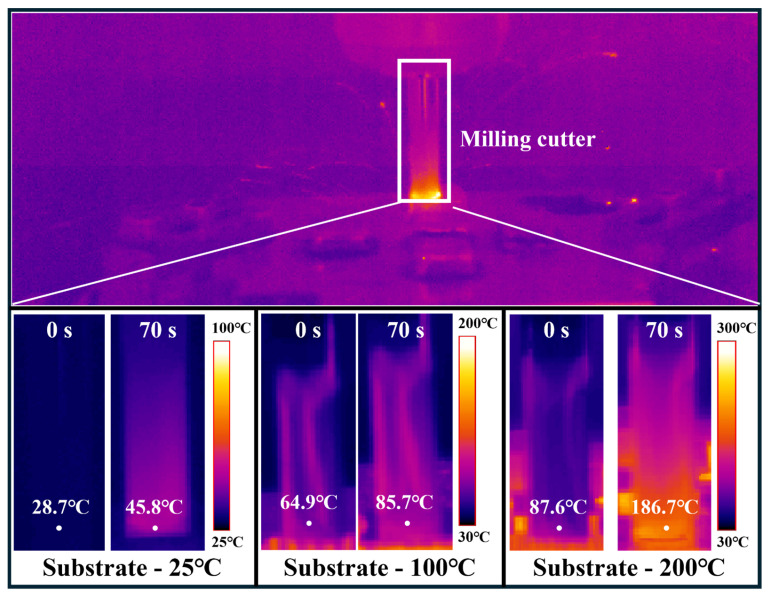
Different cooling temperatures of the substrates on the temperature of the milling cutter after 70 s of subtractive manufacturing.

**Figure 4:**
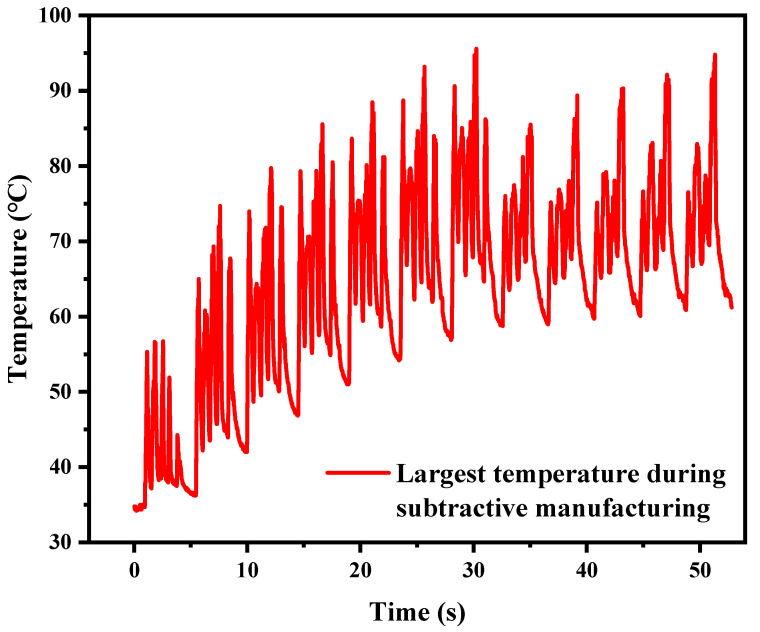
Variation tendency of the largest temperature during subtractive manufacturing when the cooling temperature was 25 °C.

The variation tendencies of the temperature characteristics are shown in Figure 3 and Figure 4. Figure 3 shows the influence of different starting temperatures on the change in tool temperature. The time required for the end-cutting process to be completed is 70 s. It is found that the higher the starting temperature of the workpiece, the higher the tool’s temperature rise. Figure 4 shows the curve of the temperature at the cutting point changing with the processing time when the initial cooling temperature is 25 °C. It can be found that the temperature at the cutting point will increase significantly in the early stage, and then will reach the thermal balance and tend to be stable. A similar phenomenon also occurs when the starting temperature of the workpiece is 100 and 200 °C. Under the combined action of increasing heat from milling and heat dissipation from the high-temperature substrate, the overall temperature of the additive manufacturing workpiece does not change significantly within 3 min of the execution of the material reduction process. This indicates that cutting at high temperatures is difficult to avoid.

### 3.3. Surface Morphology and Defects

**Figure 5:**
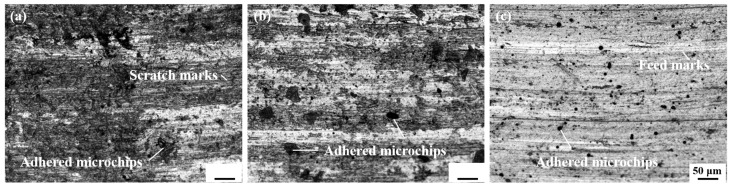
Surface morphology of cutting samples at different preheating temperatures: (**a**) 25 °C, (**b**) 100 °C, (**c**) 200 °C.

The surface morphology of turning blocks under different preheating temperatures is shown in Figure 5. The cutting surface defects mainly include feed marks, scratch marks, and adhered debris [22]. Feed marks are unavoidable defects caused by tool contact with turning material. This defect is the main cause of changes in surface roughness, which will be analyzed in detail in the next section. Scratch defects are caused by high-speed moving cutting debris and machining surface collision friction. As the preheating temperature of the substrate increases, the cutting process becomes easier under the influence of the thermal softening effect of the substrate. The interaction between the debris and the substrate weakens, and the scratches gradually disappear. Moreover, the generation of adhesive debris is due to the accumulation of debris in the tool gap during the cutting process, which falls off and adheres to the surface of the workpiece under pressure and friction in a high-temperature environment, causing a serious impact on the surface quality. As shown in Figure 5a, many irregular adhesive debris defects appeared on the sample’s surface at 25 °C. As the preheating temperature increases, the number and size of these adhesion defects decrease, and their shape gradually becomes regular. This is because, at higher temperatures, the thermal softening effect of the sample improves the shaping of the surface to be processed, reducing the degree of shear slip damage during the turning process. Debris tends to fall off in continuous strips, resulting in a significant reduction in the amount of debris adhering to the surface of the sample [25].

### 3.4. Surface Roughness

**Figure 6:**
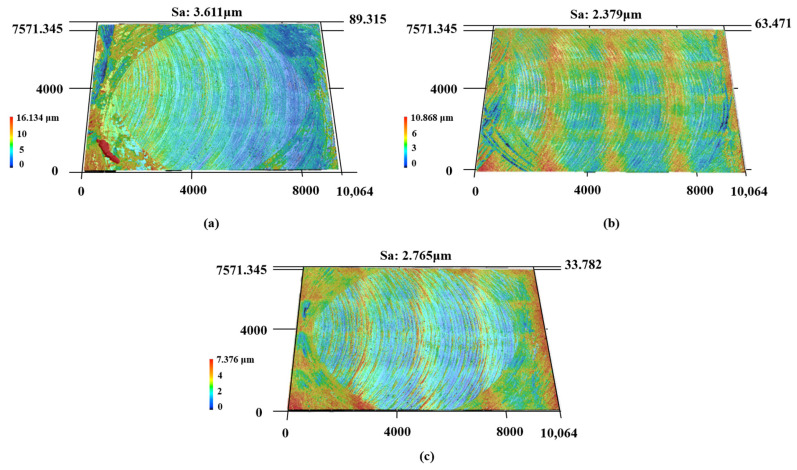
Surface roughness of cutting samples under different temperatures: (**a**) 25 °C, (**b**) 100 °C, (**c**) 200 °C.

During the cutting process, the high-frequency vibration of the milling cutter in contact with the sample block, the strong frictional force between the interfaces, and the plastic deformation directly affect the milling surface morphology, thereby affecting the surface roughness of the workpiece. The surface morphology of the milled samples at different cooling temperatures is shown in Figure 6. At 25 °C, the surface of the sample is uneven, with local protrusions and ripples. This is because the machined surface exhibits plastic protrusions and deformation rebound under high-speed cutting, causing the tool to recut the already machined surface [26]. As the preheating temperature of the substrate increases, the machined surface undergoes a thermal softening effect, and the softened surface is more easily broken during the friction and shear extrusion of the cutting tool, thereby forming a good surface morphology and lower roughness [27]. However, at excessively high temperatures, the frictional heat generated by high-speed cutting and the cooling temperature of the sample cause some materials to melt and adhere to the milling cutter head, resulting in damage to the surface quality. With an increase in preheating temperature, the surface roughness decreases first and then increases, which proves the above conclusion. The surface roughness is caused by the geometric height difference of the relative motion between the cutting head and the workpiece, plastic deformation, and tool oscillation. The preheating temperature mainly affects the plastic deformation of the surface. At a preheating temperature of 100 °C, the surface roughness reaches a minimum of 2.379 μm.

To investigate whether temperature has a significant difference in the surface quality, the surface roughness of the samples corresponding to the three preheating temperatures was measured (Table 4) to conduct ANOVA. F_0.05_ (2,12) = 692.22 > 3.885294. The results suggest that the effect of temperature on surface roughness is significant.

### 3.5. Chip and Interface Microstructure

**Figure 7:**
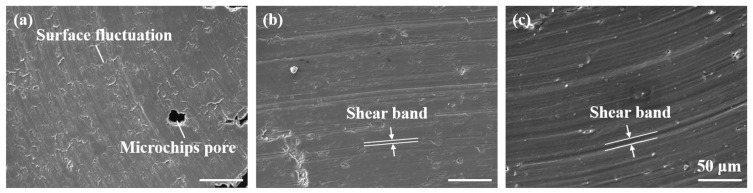
The microstructure of chips under different preheating temperatures: (**a**) 25 °C, (**b**) 100 °C, (**c**) 200 °C.

Temperature is the main factor affecting the surface morphology of cutting debris. Figure 7 shows the microstructure of cutting debris at different preheating temperatures. At 25 °C, microchip pores occur in the debris (Figure 7a), which is due to the large shear deformation during the milling process causing the chips to peel off under high cutting heat, resulting in the release of heat. Moreover, due to the severe thermoplastic instability of the material during the cutting process, the degree of slip on the machined surface is large, resulting in surface fluctuations of debris, which also affects the surface roughness of the machined surface [28]. As the preheating temperature of the substrate increases, continuous shear bands appear on the free surface. This type of adiabatic shear band originates from a rapid plastic deformation process, which generates heat that cannot be dissipated in time, limiting the shear action to the cutting surface. At a preheating temperature of 200 °C, the thermal softening effect of the processed sample promotes smoother thermal deformation of the substrate, resulting in less shear slip deformation and uniform shear bands on the free surface of the debris.

**Figure 8:**
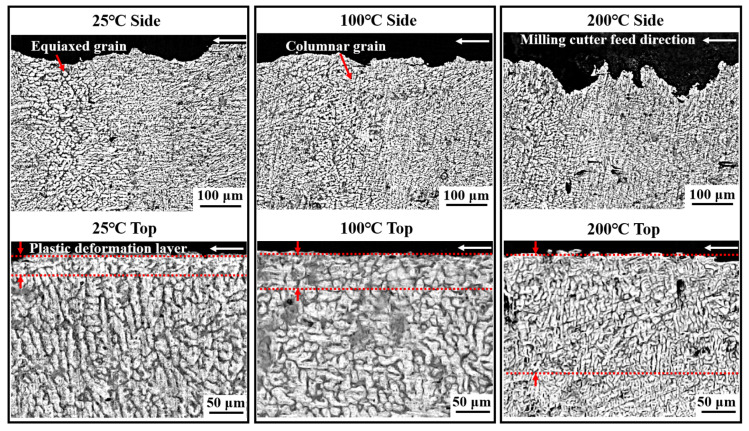
Microstructure of sample interface.

Figure 8 shows the microstructure of the cutting boundary, where the side is the edge parallel to the axis of the wear tool and the top is the edge perpendicular to the axis of the wear tool. The microstructures on the side of the sample are columnar-equiaxed bimodal microstructures common in laser additive manufacturing samples [29]. It is worth noting that at 25 °C, the side surface is uneven. When the preheating temperature is raised to 100 °C, the quality of the side surface improves, which then deteriorates again under 200 °C. This may be because, at a preheating temperature of 100 °C, the substrate is more prone to shear deformation under the effect of thermal softening, resulting in higher surface quality than at 25 °C. However, under 200 °C, the high temperature and high pressure during the milling process cause the material to adhere to the surface of the milling cutter, which has an adverse effect on the surface quality of the side edges. On the top edge, it was found that the surface grains were twisted and deformed [30]. This deformation layer is caused by the huge shear force generated by the contact between the milling cutter and the substrate during the milling process, which causes the grains on the surface of the machined surface to deflect. As the preheating temperature increases, the width of the plastic deformation layer gradually increases. This is due to the high temperature causing thermal softening of the material surface. When in contact with the milling cutter, a plowing effect is generated on the machined surface, which becomes more significant with increasing cutting speed and temperature [31,32]. The maximum thickness of the plastic deformation layer reaches 175 μm under the preheating temperature of 200 °C.

### 3.6. Tool Vibration and Wear

**Figure 9:**
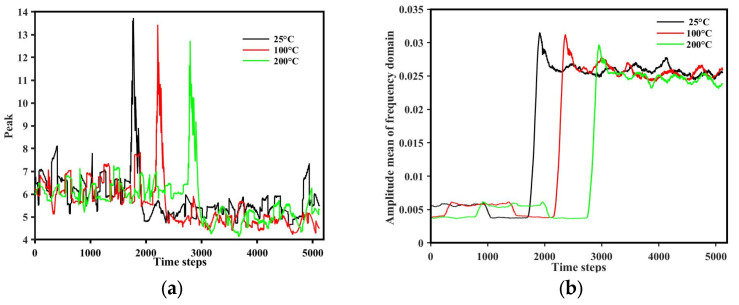
Tool vibration comparison: (**a**) peak value and (**b**) amplitude mean of frequency domain during milling.

The results of vibration testing during the cutting tool process are shown in Figure 9. It can be seen that the increase in substrate temperature reduces the amplitude of vibration at the starting point of milling. The peak value when the tool contacts the substrate decreases with an increase in temperature, as shown in Figure 9. During the milling process, the peak value and the amplitude mean of the frequency domain of the 25 °C substrate temperature are larger. It means that the hardness of the substrate decreases with an increase in the substrate temperature. However, the difference in the tool vibrations between the substrate temperatures of 100 and 200 °C is not obvious.

As the sample temperature increases, the matrix hardness decreases, and the tool cutting becomes easier, which is conducive to maintaining process stability and reducing tool wear. Zhang et al. [33] proposed a relationship between material hardness and critical shear fracture stress based on the Tresca criterion and von Mises criterion [34].

$${2\tau }_{0}=k_{1}\sigma_{y}$$

$$H_{v}=k_{2}\sigma$$

where $\tau_{0}$ is the critical shear fracture stress, $k_{1}$ and $k_{2}$ are constant, $\sigma_{y}$ is the yielding stress, $H_{v}$ is the hardness, and σ is the strength (yield strength or ultimate tensile strength).

Hardness is a temperature-dependent mechanical property. The indentation test is an effective method to evaluate the mechanical properties of materials. With the development of indentation testing methods at micro and nano scales, it has been shown that plastic deformation based on the indentation size effect (ISE) [35] is mainly caused by the plastic strain energy dissipation at the intended surface [18,19].

$$H_{v}=\frac{\sigma_{y}}{{S/V}^{2/3}}\cdot \frac{1}{{3hR}^{1/3}}$$

where *S* and *V* represent the contact surface and the plastic volume, *h* represents the indentation depth, and *R* is the radius of the indenter. Further, a theoretical temperature-dependent hardness model (TDH) was established [36].

$$H_{v}\left(T\right)=H_{v}\left(T_{0}\right)\cdot {\left[\frac{E(T)}{E(T_{0})}\cdot \left(1-\frac{T-T_{0}}{T_{m}-T_{0}}\right)\right]}^{1/2}$$

where $H_{v}\left(T\right)$ and $H_{v}\left(T_{0}\right)$ are the hardness of the material at temperatures *T* and $T_{0}$ (25 °C). E(T) and $E(T_{0})$ are the Young’s modulus at different temperatures, and $T_{m}$ is the melting temperature. The quantitative relationship between cutting force and temperature was then established.

$$\tau =k{\cdot H}_{v}\left(T_{0}\right)\cdot {\left[\frac{E(T)}{E(T_{0})}\cdot \left(1-\frac{T-T_{0}}{T_{m}-T_{0}}\right)\right]}^{1/2}$$

**Figure 10:**
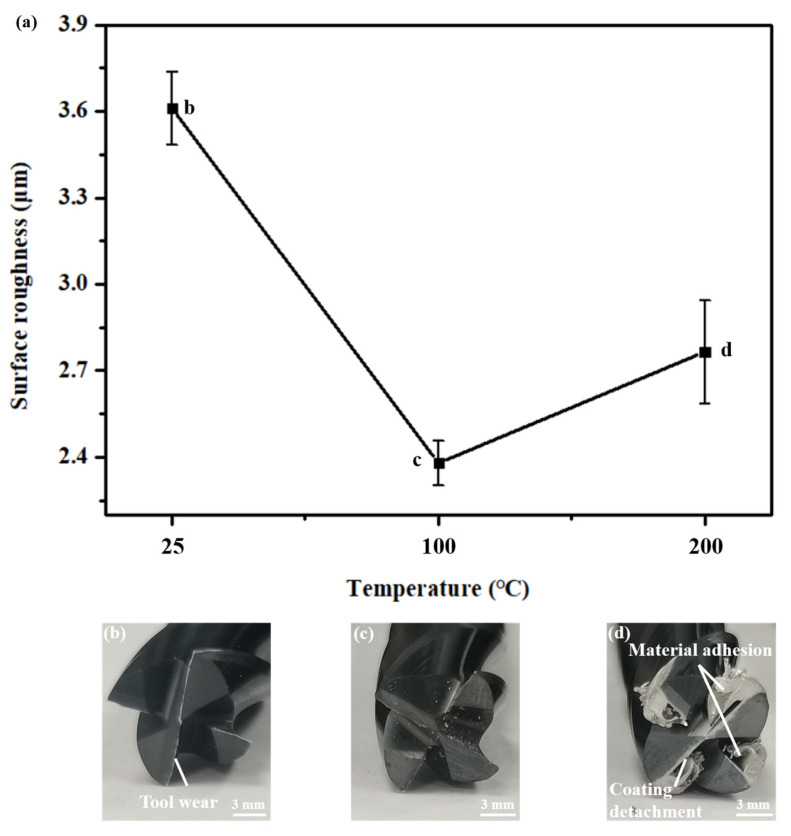
Effect of temperature on (**a**) surface roughness and (**b**–**d**) tool wear.

In the process of subtractive manufacturing, tool wear directly affects the shear friction between the cutting head and the surface to be machined, thereby affecting the surface morphology of the subtractive material. Figure 10 shows tool wear and surface roughness at different preheating temperatures. Tool head damage includes the coating material falling off, substrate material adhesion, tool base wear, etc.

At a preheating temperature of 25 °C, the stress concentration at the tip of the tool induces significant shear slip between the workpiece and the tool head. As a result, the tool tip exhibits noticeable wear and a dull appearance. Correspondingly, the surface roughness of the machined workpiece is observed to be substantial. As the preheating temperature is elevated to 100 °C, the surface tip of the blade remains largely undamaged, with only minor debris adhering. Consequently, the surface quality improves markedly, and the surface roughness is effectively minimized. When the preheating temperature is 200 °C, the bonding wear between the tool and the substrate material causes the tool coating to bond with the tool substrate material. Furthermore, the elevated temperature and pressure during the cutting process result in the accumulation of continuous debris within the gaps of the cutting heads, thereby increasing the thickness of the material bonding layer along the side edges of the tool [23]. This accumulation adversely affects normal cutting operations. Simultaneously, the surface roughness of the sample experiences a slight increase, and the surface displays ridge-like ripples. This phenomenon can be attributed to the adhesion of chips to the tool, which leads to fluctuations in cutting force and induces a wavy stress state on the surface [37,38]. This results in pronounced wavy features on the machined surface, as depicted in Figure 6c.

## 4. Discussion

### 4.1. Temperature Change Behavior in Additive–Subtractive Manufacturing

Because the LDED used in laser additive manufacturing has a high energy input characteristic, the temperature of the workpiece will rise sharply during the processing. This is followed by a rapid temperature drop due to the rapid solidification. However, there is a bottleneck effect in this temperature drop, which can be called a turning point temperature, as shown in Figure 2, where there is a rapid temperature gradient at a turning point, after which the cooling rate drops significantly. As the number of layers increases, the turning temperature will gradually rise with the heat accumulation phenomenon. The model E_all_ = E_in_ − E_out_ was established, where E_all_ is the accumulated heat energy of the sample, E_in_ is the laser input energy, and E_out_ is the heat lost. E_out_ includes heat conduction, heat radiation, and convective heat loss, of which the influence of heat conduction on heat loss is most obvious [39]. Therefore, the gradual increase in the turning point temperature is mainly affected by heat conduction. When the laser processing ends, the heat begins to flow from the deposited layer to the base plate, then from the base plate to the lower work platform in turn, and the heat conduction speed is determined by the temperature difference ∆T. The increase in the turning point temperature also means an increase in heat accumulation, which will have a counteracting effect on the next stage of the deposition process. For subtraction manufacturing, the main heat comes from cutting heat, which will cause both the sample and the cutting tool to heat up. Figure 4 shows that compared to additive processes, this part has a very limited impact on temperature, and the heat accumulation model can be changed to E_all_ = E_laserin_ − E_out_ + E_millingin_. This effect also needs to be regulated in actual processing to avoid the failure of pre-designed heat control. Overall, the heat accumulation change pattern can be adjusted by adjusting the additive process, subtraction process, and heat dissipation conditions.

### 4.2. Intervention Time of the Milling During the ASM

According to the results of Section 3.3, Section 3.4, Section 3.5 and Section 3.6, the intervention time of the material reduction process in the process of adding and reducing aluminum alloy can be evaluated from the aspects of processing quality and impact on the tool. From the perspective of processing quality, because the aluminum alloy itself has a low melting point, the friction heat generated by high-speed cutting and the cooling temperature of the sample make some materials melt and adhere to the milling head, resulting in the inability to continue milling, resulting in the phenomenon of sticking, so 300 °C heating cannot complete the material reduction process. At 25, 100, and 200 °C, the material reduction process can be completed, and because the temperature rises, the thermal softening effect is formed and the hardness of the sample is reduced, so the overall processing is smoother, the chips are more coherent, and the surface damage is less. A similar effect is observed for roughness, with local bumps and ripples on the surface of the sample at 25 °C. This is because the plastic protruding and deformation rebounds appear on the machined surface under high-speed cutting, causing the tool to recut the already machined surface. With an increase in the preheating temperature of the substrate, a thermal softening effect occurs on the machined surface, and the softened surface is more prone to fracture during the friction and shear extrusion process of the tool, thus forming a good surface topography and lower roughness. When the preheating temperature is 200 °C, the surface roughness reaches 8.926 μm. There are some differences between side milling and top milling. The high temperature and high pressure in the milling process cause the material to adhere to the milling cutter surface on the side surface, making it difficult to remove chips, as shown in Figure 10c, and thus adversely affecting the surface quality of the side, as shown in Figure 8. Therefore, the optimal temperature is about 100 °C. In addition, a significant difference in the thickness of the plastic deformation layer was found, with the temperature increasing by 200 degrees; the maximum thickness of the plastic deformation layer could reach 175 μm. The grain at the plastic deformation layer is finer, more slender, and more prone to defects. Therefore, in the case of the additive composite process, it is necessary to focus on the influence of the plastic deformation layer on the overall quality. If the quality can be guaranteed, the plastic deformation layer may be used for a performance enhancement effect.

For the tool, the temperature increase can soften the aluminum sample and reduce the tool vibration, but the excessive temperature drop directly causes the aluminum to adhere to the milling cutter, resulting in processing failure and tool damage. Although processing can occur at 200 degrees, there are still chips adhering to the milling cutter that are difficult to discharge, resulting in a reduction in the quality of side processing. This inability to stabilize the chip removal phenomenon will also lead to tool wear fixture; therefore, out of the starting temperatures of 25, 100, and 200 °C, the minimum tool wear occurs at 100 °C. Therefore, there should be an interval range of about 100 °C at the time of cutting material intervention, which has a better effect on the quality of the cutting material and tool damage.

## 5. Conclusions

(1) During the cooling process of LDED, there is a turning point temperature that has an important impact on the temperature change. After the turning point temperature, the temperature will change from a sharp decline to a slow decline. The continuous heat accumulation between the substrate and the working environment makes the turning temperature gradually rise.

(2) An increase in the cooling temperature will result in a process where milling quality initially increases and then decreases. The increase is due to the softening of aluminum, which causes a decrease in hardness and vibration. The decrease is due to the high-temperature aluminum adhering to the tool. Better milling quality also means less tool wear.

(3) An increase in the cooling temperature results in an increase in the thickness of the adiabatic shear band to 175 μm, which may have an impact on the quality of the ASM processes and needs to be avoided or effectively utilized.

(4) In this work, for the aluminum alloy, the optimal milling temperature should be around 100 °C. In the design of the process parameters of ASM, it is necessary to consider how to set the initial cooling temperature of the subtraction process within this range, for example, by using a heat dissipation plate or low-temperature cooling.

(5) This study offers guidance for subtractive manufacturing during the cooling of additive manufacturing samples. Future research should examine the correlation between cooling temperatures of medium/large components in industrial processing and the quality of material reduction, alongside conducting long-term experiments to assess tool durability. In addition, industrial applications also need to consider the coupling effect of subtractive manufacturing process parameters and cooling temperatures.

## Figures and Tables

**Table 1:** Chemical composition (wt.%) of AlSi7Mg powder.

Element	Si	Mg	Cu	Mn	Zn	Ni	Fe	TiB_2_	Al
AlSi7Mg	6.50~7.50	0.90~1.50	≤0.10	≤0.10	≤0.10	≤0.10	≤0.10	1.50~2.50	Balanced

**Table 2:** Process parameter design for additive manufacturing.

Laser power (W)	Scanning speed (mm/s)	Powder feed rate (g/min)	Z-axis increment (mm)	Hatch spacing (mm)
1600	18	3	0.3	1.6
Shielding gas rate (L/h)	Carrier gas rate (L/h)	Sample dimension (mm × mm × mm)	Residence time between layers (s)	Residence time between tracks (s)
600	240	20 × 20 × 10	30	0

**Table 3:** Process parameter design for subtractive manufacturing.

Process Parameter	Value
Initial cooling temperature (°C)	25, 100, 200, 300
Feed speed (mm/min)	1000
Spindle speed (r/min)	3000
milling depth (mm/layer)	0.3

**Table 4:** Surface roughness at different preheating temperatures.

Temperature (°C)	25	100	200
Surface roughness (μm)	3.617	2.413	2.729
	3.584	2.335	2.678
	3.682	2.359	2.740
	3.619	2.382	2.857
	3.555	2.406	2.822

## Data Availability

The original contributions presented in the study are included in the article, further inquiries can be directed to the corresponding author.

## References

[1] Lu H., Wu L., Wei H., Cai J., Luo K., Xu X., Lu J. (2022). Microstructural evolution and tensile property enhancement of remanufactured Ti6Al4V using hybrid manufacturing of laser directed energy deposition with laser shock peening. Addit. Manuf..

[2] Su Y., Xu G., Xu X., Zhang H., Luo K., Lu J. (2024). Melt pool control-assisted additive manufacturing of thin-walled parts. Int. J. Mech. Sci..

[3] Tang Z.-J., Liu W.-W., Wang Y.-W., Saleheen K.M., Liu Z.-C., Peng S.-T., Zhang Z., Zhang H.-C. (2020). A review on in situ monitoring technology for directed energy deposition of metals. Int. J. Adv. Manuf. Technol..

[4] Feldhausen T., Raghavan N., Saleeby K., Love L., Kurfess T. (2021). Mechanical properties and microstructure of 316L stainless steel produced by hybrid manufacturing. J. Mater. Process. Technol..

[5] Lv J., Liang Y., Xu X., Xu G., Zhang H., Lu H., Luo K., Cai J., Lu J. (2024). Performance-control-orientated hybrid metal additive manufacturing technologies: State of the art, challenges, and future trends. Int. J. Extrem. Manuf..

[6] Weiss L., Prinz F., Neplotnik G., Padmanabhan K., Schultz L., Merz R. (1997). Shape deposition manufacturing of wearable computers. Eng. Comput. Sci..

[7] Xu X., Du J., Lu H., Su Y., Xing F., Luo K., Lu J. (2024). High-performance functional coatings manufactured by integrated extremely high-speed-rate laser directed energy deposition with interlayer remelting. Int. J. Mach. Tools Manuf..

[8] Tian S., Wang Z., Wang Z., Luo K., Lu J. (2023). The mechanism of anisotropic micro-milling properties in additively manufactured Ti-6Al-4V alloy. J. Mater. Process. Technol..

[9] Djogo G., Li J., Ho S., Haque M., Ertorer E., Liu J., Song X., Suo J., Herman P.R. (2019). Femtosecond laser additive and subtractive micro-processing: Enabling a high-channel-density silica interposer for multicore fibre to silicon-photonic packaging. Int. J. Extrem. Manuf..

[10] Butt J., Hewavidana Y., Mohaghegh V., Sadeghi-Esfahlani S., Shirvani H. (2019). Hybrid manufacturing and experimental testing of glass fiber enhanced thermoplastic composites. J. Manuf. Mater. Process..

[11] Du W., Bai Q., Zhang B. (2016). A novel method for additive/subtractive hybrid manufacturing of metallic parts. Procedia Manuf..

[12] Xie Y., Tong J., Fu Y., Sheng Z. (2020). Machining scheme of aviation bearing bracket based on additive and subtractive hybrid manufacturing. J. Mech. Sci. Technol..

[13] Chernovol N., Sharma A., Tjahjowidodo T., Lauwers B., Van Rymenant P. (2021). Machinability of wire and arc additive manufactured components. CIRP J. Manuf. Sci. Technol..

[14] Alexeev V., Balyakin A., Khaimovich A. (2024). Influence of the direction of selective laser sintering on machinability of parts from 316L steel. Proceedings of IOP Conference Series: Materials Science and Engineering.

[15] Bai Y., Lee Y.J., Zhao C., Yan Q., Guo Y., Shang Y., Wang H. (2023). Unique cellular microstructure-enabled hybrid additive and subtractive manufacturing of aluminium alloy mirror with high strength. J. Mater. Process. Technol..

[16] Jeon Y., Pfefferkorn F. (2008). Effect of laser preheating the workpiece on micro end milling of metals. J. Manuf. Sci. Eng..

[17] Hedberg G., Shin Y., Xu L. (2015). Laser-assisted milling of Ti-6Al-4V with the consideration of surface integrity. Int. J. Adv. Manuf. Technol..

[18] Grzesik W. (2018). Hybrid additive and subtractive manufacturing processes and systems: A review. J. Mach. Eng..

[19] Li S., Zhang B., Bai Q. (2020). Effect of temperature buildup on milling forces in additive/subtractive hybrid manufacturing of Ti-6Al-4V. Int. J. Adv. Manuf. Technol..

[20] Lee I., Bajpai V., Moon S., Byun J., Lee Y., Park H.W. (2015). Tool life improvement in cryogenic cooled milling of the preheated Ti–6Al–4V. Int. J. Adv. Manuf. Technol..

[21] Tang Z., Wei Q., Gao Z., Yang H., Wang A., Wan L., Luo C., Wu Y., Wang H., Wang H. (2023). 2000W blue laser directed energy deposition of AlSi7Mg: Process parameters, molten pool characteristics, and appearance defects. Virtual Phys. Prototyp..

[22] Li C., Liu X., Xu M., Chen J., Li S., Li P., Ko T.J. (2023). Formation Mechanism and Adhesion Evaluation of Debris in Ti–6Al–4V Alloy Turning. Int. J. Precis. Eng. Manuf.-Green Technol..

[23] Puvanesan M., Rahman M., Najiha M., Kadirgama K. (2014). Experimental investigation of minimum quantity lubrication on tool wear in aluminum alloy 6061-t6 using different cutting tools. Int. J. Automot. Mech. Eng..

[24] Wang A., Wei Q., Tang Z., Oliveira J.P., Leung C.L.A., Ren P., Zhang X., Wu Y., Wang H., Wang H. (2024). Effects of hatch spacing on pore segregation and mechanical properties during blue laser directed energy deposition of AlSi10Mg. Addit. Manuf..

[25] Liang X., Liu Z., Yao G., Wang B., Ren X. (2019). Investigation of surface topography and its deterioration resulting from tool wear evolution when dry turning of titanium alloy Ti-6Al-4V. Tribol. Int..

[26] Huang X., Bai Q., Li Y.T., Zhang B. (2017). Machining finish of titanium alloy prepared by additive manufacturing. Appl. Mech. Mater..

[27] Xavierarockiaraj S., Kuppan P. (2014). Investigation of cutting forces, surface roughness and tool wear during Laser assisted machining of SKD11Tool steel. Procedia Eng..

[28] Li C., Xu M., Yu Z., Huang L., Li S., Li P., Niu Q., Ko T.J. (2020). Electrical discharge-assisted milling for machining titanium alloy. J. Mater. Process. Technol..

[29] Sun T., Chen J., Wu Y., Wang M., Fu Y., Wang H., Wang H. (2022). Achieving excellent strength of the LPBF additively manufactured Al–Cu–Mg composite via in-situ mixing TiB2 and solution treatment. Mater. Sci. Eng. A.

[30] Du S., Jiang Z., Zhang D., Wang Z., Li N. (2015). Microstructure of plastic deformation layer on grinding surface of GH4169 alloy. J. Mech. Eng..

[31] Habrat W., Krupa K., Markopoulos A.P., Karkalos N.E. (2021). Thermo-mechanical aspects of cutting forces and tool wear in the laser-assisted turning of Ti-6Al-4V titanium alloy using AlTiN coated cutting tools. Int. J. Adv. Manuf. Technol..

[32] Li G., Chandra S., Rashid R.A.R., Palanisamy S., Ding S. (2022). Machinability of additively manufactured titanium alloys: A comprehensive review. J. Manuf. Process..

[33] Zhang P., Li S., Zhang Z. (2011). General relationship between strength and hardness. Mater. Sci. Eng. A.

[34] Tabor D. (2000). The Hardness of Metals.

[35] Liu J.-X., Shen X.-Q., Wu Y., Li F., Liang Y., Zhang G.-J. (2020). Mechanical properties of hot-pressed high-entropy diboride-based ceramics. J. Adv. Ceram..

[36] Liu Y., Wang R., Wan Y., Zhou S., Cai H., Gu M., Li D., Li W. (2022). Temperature-dependent hardness model of high-temperature materials can account for indentation size effect. Int. J. Refract. Met. Hard Mater..

[37] Mabrouki T., Girardin F., Asad M., Rigal J.-F. (2008). Numerical and experimental study of dry cutting for an aeronautic aluminium alloy (A2024-T351). Int. J. Mach. Tools Manuf..

[38] Sun S., Brandt M., Dargusch M.S. (2009). Characteristics of cutting forces and chip formation in machining of titanium alloys. Int. J. Mach. Tools Manuf..

[39] Tang Z.-J., Liu W.-W., Zhu L.-N., Liu Z.-C., Yan Z.-R., Lin D., Zhang Z., Zhang H.-C. (2021). Investigation on coaxial visual characteristics of molten pool in laser-based directed energy deposition of AISI 316L steel. J. Mater. Process. Technol..

